# Discrepancies in listed adverse drug reactions in pharmaceutical product information supplied by the regulatory authorities in Denmark and the USA

**DOI:** 10.1002/prp2.38

**Published:** 2014-04-22

**Authors:** Robert Eriksson, Lise Aagaard, Lars Juhl Jensen, Liza Borisova, Dorte Hørlück, Søren Brunak, Ebba Holme Hansen

**Affiliations:** 1 Department of Disease Systems Biology, NNF Center for Protein Research, Faculty of Health and Medical Sciences, University of CopenhagenBlegdamsvej 3B, DK-2200, Copenhagen, Denmark; 2 Center for Biological Sequence Analysis, Department of Systems Biology, Technical University of DenmarkKemitorvet, DK-2800, Lyngby, Denmark; 3 Institute of Public Health, Clinical Pharmacology, Faculty of Health Sciences, University of Southern DenmarkJ.B. Winsløws Vej 19, DK-5000, Odense, Denmark; 4 Danish Pharmacovigilance Research Project (DANPREP)Copenhagen, Denmark; 5 Department of Pharmacy, Faculty of Health and Medical Sciences, University of CopenhagenUniversitetsparken 2, DK-2100, Copenhagen, Denmark

**Keywords:** Adverse drug reaction, Denmark, drug approval, drug labeling, drug legislation, European Medicines Agency, Food and Drug Administration, Product information, Regulatory affairs, Summary of Product Characteristics, United States

## Abstract

Pharmaceutical product information (PI) supplied by the regulatory authorities serves as a source of information on safe and effective use of drugs. The objectives of this study were to qualitatively and quantitatively compare PIs for selected drugs marketed in both Denmark and the USA with respect to consistency and discrepancy of listed adverse drug reaction (ADR) information. We compared individual ADRs listed in PIs from Denmark and the USA with respect to type and frequency. Consistency was defined as match of ADRs and of ADR frequency or match could not be ruled out. Discrepancies were defined as ADRs listed only in one country or listed with different frequencies. We analyzed PIs for 40 separate drugs from ten therapeutic groups and assigned the 4003 identified ADRs to System Organ Classes (Medical Dictionary for Regulatory Activities [MedDRA] terminology). Less than half of listed ADRs (*n* = 1874; 47%) showed consistency. Discrepancies (*n* = 2129; 53%) were split into ADRs listed only in the USA (*n* = 1558; 39%), ADRs listed only in Denmark (*n* = 325; 8%) and ADRs listed with different frequencies (*n* = 246; 6%). The majority of listed ADRs were of the type “gastrointestinal disorders” and “nervous system disorders”. Our results show great differences in PIs for drugs approved in both Denmark and the USA illuminating concerns about the credibility of the publicly available PIs. The results also represent an argument for further harmonization across borders to improve consistency between authority-supplied information.

## Introduction

Access to accurate and up-to-date information about safe use of drugs is important for prescribers and dispensers. The official product information (PI) serves as an authoritative source of information (European Commission 2009; Food and Drug Administration 2013), which marketing authorization holders (MAHs) are obliged to update if new information is discovered. Inconsistencies in this authority-supplied information would cause healthcare professionals to base prescribing decisions on different premises and unequal grounds. This is not only problematic in the initiation of a drug treatment but also influences any investigation of suspected adverse drug reactions (ADRs).

Drug information from different resources that is available to healthcare professionals has previously been studied (Dunne et al. 1973; Silverman 1976; Alloza and Lasagna 1983; Bawazir et al. 1991; Reggi et al. 2003; Malinowski et al. 2008; Sawalha et al. 2008; Sasich et al. 2009; Sukkari et al. 2012). Few articles have compared safety information listed in the PIs across countries and regulatory systems. Garbe and Andersohn (2007) assessed whether contraindications added to PIs from the USA were also added to the German PIs. They found that less than half of the contraindications added to the PI in the USA had been fully incorporated and listed in Germany. Nieminen et al. (2005) compared PIs for biopharmaceuticals approved in the EU and the USA. The authors reported that 14 of 32 PIs had the same degree of detail, 15 were less detailed, and three more detailed in the EU. Shimazawa and Ikeda (2013) compared safety information in PIs from the USA, the United Kingdom, and Japan by comparing the number of words in sections concerning safety, and they concluded that substantial differences in safety information existed. Kesselheim et al. (2013) applied a similar method. The PIs of 20 drugs from the USA, Canada, the United Kingdom, and Australia were analyzed by comparing the number of ADRs present in each country, and large differences were found. A recap (Prescrire 2013) of cases in 2012 further provided incentive to study the problem, by underlining multiple aspects concerning drug safety issues.

The objectives of the present study was to qualitatively and quantitatively compare PIs for selected drugs marketed in both Denmark and the USA with respect to consistency and discrepancy of listed ADR information. We compared the individual listed ADRs of each drug with respect to type and frequency across a broad range of substances and MAHs.

## Method

### Selection of drugs

The basis for this analysis was all therapeutic groups at the second Anatomical Therapeutic Chemical (ATC) classification system level (WHO Collaborating Centre for Drug Statistics Methodology 2012). We used defined daily dose (DDD) from the Danish population as a cut-off. The threshold was set to minimum 50 sold DDD per 1000 inhabitants per day in year 2010 (Statens Serum Institut 2012). We excluded combination drugs, drugs not marketed by the original MAH in both Denmark and the USA and drugs not available on the authorities’ websites. The authorities’ websites were used to determine if the drugs were currently marketed in Denmark (Lægemiddelstyrelsen 2012a) and the USA (Food and Drug Administration 2012). We required the PIs to be issued by the original MAH and the highest marketed strength in Denmark and the USA to be the same.

### Product information

In the EU, ADR information is presented in the document called Summary of Product Characteristics (SPC) (European Commission 2009) and in the USA, the information can be found in the drug’s label (Food and Drug Administration 2013). In this article, we apply the terminology PI for data from both countries. The PIs were retrieved from the website of the respective regulatory agencies between February and April 2012. Danish PIs were available from either the Danish Medicines Agency (DMA) [now Danish Health and Medicines Authority] (Læge-middelstyrelsen 2012b) or the European Medicines Agency (EMA) (European Medicines Agency 2012) depending on if the drug was approved through the national or the centralised EU procedure. FDA PIs were retrieved from Drugs@FDA (Food and Drug Administration 2012).

### Data collection and category assignment

For each included drug, we manually extracted all information about ADRs and frequencies listed in the PIs. The same type of ADR was only counted once (e.g., *thirst* and *polydipsia*). The ADRs were assigned into the corresponding Medical Dictionary for Regulatory Activities (MedDRA) (Brown 2007) system organ class (SOC) or a separate category for all data not possible to assign a SOC. Information about seriousness of the ADRs listed in the PIs was scarce and, therefore, this parameter was not included in this study. All data extractions and analyses were made by the fourth and fifth author and checked by the first author.

### Definition and classification of ADRs

Different terminology is applied in the countries and, therefore, we use ADR to cover both *undesirable effects* used in the EU and *adverse reactions* used in the USA. The European Commission (European Commission 2009) mandates ADR frequency information into five frequency intervals plus an additional category ‘not known’ (Table 1), whereas the FDA (Food and Drug Administration 2006) advises to group ADRs within appropriately specified frequency ranges. It is common practice that drug labels approved by the FDA contain intervals quite similar to the European intervals, but often with only four intervals. We often observed the same definitions used to describe fixed frequency intervals and we have therefore based the analysis on the ranges these definitions refer to (Table 1). Frequencies defined with a numerical share were generalized into these intervals.

**Table 1:** Definition of adverse drug reaction frequency intervals in Denmark and the USA

Frequency	European commission definition	Definition often observed in PIs from the USA	Frequency interval
≥1/10	Very common	Frequent	Higher frequency interval
≥1/100 to <1/10	Common	Frequent	Higher frequency interval
≥1/1000 to <1/100	Uncommon	Infrequent	Higher frequency interval
≥1/10,000 to <1/1000	Rare	Rare	Lower frequency interval
<1/10,000	Very rare	Rare	Lower frequency interval
Not estimated	Not known	Unknown	Lower frequency interval

We defined consistency and discrepancy of ADR information in eight categories (Table 2). Consistency was specified as one of three conditions: First, ADRs listed as occurring at the same frequency interval in both countries. Second, ADRs listed in both countries but without information about the frequency. Third, ADRs possibly listed with same frequency in both countries meaning that due to the use of different frequency intervals, consistency could not be dismissed. ADRs that were consistent under the second and third conditions were merged in our analysis. Discrepancy of ADR information was defined as different frequencies listed in the countries or only listed in one country. Difference in frequency was dichotomized into ADRs with contiguous frequencies, consisting of ADRs listed in two adjacent frequency intervals, and noncontiguous frequencies, consisting of ADRs listed in two nonadjacent frequency intervals. Information only listed in one country was separated into a higher and a lower interval (Table 1).

**Table 2:** Consistency and discrepancy of analyzed drug substances

				Consistent				Discrepant													
					
						ADR listed where corresponding frequencies cannot be dismissed		ADR listed with frequency difference				ADR only listed in Denmark				ADR only listed in the USA					
																			
		Product information updated		ADR listed with corresponding frequency				Contiguous frequency difference		Noncontiguous frequency difference		Rare, very rare or not known		Very common, common or uncommon		Rare or unknown		Frequent or infrequent		Sum	
Drug	ATC	Denmark	USA	*n*	%	*n*	%	*n*	%	*n*	%	*n*	%	*n*	%	*n*	%	*n*	%	*n*	%
Losartan^3^	C09CA01	4 January 2011	17 November 2011	5	5	16	15	0	0	7	7	2	2	4	4	50	47	23	21	107	100
Valsartan^2^	C09CA03	25 January 2012	18 January 2012	17	22	12	15	0	0	9	11	11	14	6	8	4	5	20	25	79	100
Irbesartan^1^	C09CA04	10 October 2011	18 January 2012	23	25	2	2	0	0	5	5	2	2	1	1	51	55	8	9	92	100
Telmisartan^1^	C09CA07	9 March 2012	19 January 2012	31	32	4	4	1	1	18	18	1	1	0	0	0	0	43	44	98	100
Simvastatin^3^	C10AA01	21 October 2011	6 June 2011	30	54	10	18	7	13	0	0	0	0	0	0	0	0	9	16	56	100
Atorvastatin^3^	C10AA05	2 January 2012	17 June 2009	19	30	21	33	0	0	1	2	6	10	15	24	0	0	1	2	63	100
Rosuvastatin^2^,^3^	C10AA07	9 June 2010	19 November 2011	13	57	6	26	0	0	0	0	0	0	0	0	3	13	1	4	23	100
Fluvastatin	C10AA04	5 December 2011	17 June 2011	22	49	1	2	2	4	0	0	1	2	0	0	0	0	19	42	45	100
Dipyridamole	B01AC07	25 November 2008	1 August 2005	10	56	2	11	0	0	0	0	0	0	2	11	4	22	0	0	18	100
Clopidogrel^1^,^3^	B01AC04	4 January 2011	20 December 2012	43	68	8	13	0	0	0	0	1	2	10	16	1	2	0	0	63	100
Dalteparin	B01AB04	28 July 2010	22 October 2010	5	36	5	36	0	0	0	0	1	7	1	7	2	14	0	0	14	100
Ticagrelor^1^,^2^,^3^	B01AC24	7 January 2011	20 July 2011	2	4	22	47	0	0	5	11	4	9	5	11	0	0	9	19	47	100
Fentanyl^3^	N02AB03	5 May 2011	31 July 2009	59	40	0	0	2	1	7	5	2	1	26	18	0	0	50	34	146	100
Sumatriptan	N02CC01	5 August 2010	21 July 2010	24	32	12	16	0	0	3	4	0	0	1	1	21	28	15	20	76	100
Clonidine	N02CX02	14 July 2011	7 April 2010	9	15	25	41	0	0	0	0	0	0	0	0	27	44	0	0	61	100
Eletriptan^2^,^3^	N02CC06	2 June 2008	13 April 2011	72	38	0	0	0	0	14	7	0	0	0	0	89	47	14	7	189	100
Citalopram	N06AB04	10 January 2012	12 August 2011	69	34	17	8	1	0	6	3	4	2	5	2	51	25	51	25	204	100
Escitalopram^2^	N06AB10	13 February 2011	12 May 2011	57	52	6	6	0	0	7	6	2	2	2	2	8	7	27	25	109	100
Sertraline^3^	N06AB06	24 January 2012	9 August 2011	134	42	80	25	0	0	21	7	19	6	9	3	37	12	20	6	320	100
Venlafaxine	N06AX16	28 February 2012	1 June 2010	67	18	13	4	0	0	9	2	4	1	2	1	171	46	105	28	371	100
Duloxetine^1^,^2^,^3^	N06AX21	30 August 2011	2 September 2011	85	61	5	4	0	0	15	11	7	5	10	7	7	5	10	7	139	100
Amlodipine	C08CA01	22 December 2011	31 October 2011	52	48	0	0	11	10	4	4	5	5	2	2	15	14	19	18	108	100
Felodipine	C08CA02	10 January 2012	7 June 2004	9	12	45	58	0	0	7	9	5	6	5	6	0	0	6	8	77	100
Nifedipine	C08CA05	15 December 2011	27 September 2011	20	25	5	6	3	4	4	5	11	14	12	15	12	15	14	17	81	100
Budesonide	R03BA02	10 January 2012	20 August 2007	19	38	0	0	3	6	0	0	4	8	1	2	0	0	23	46	50	100
Tiotropium^2^	R03BB04	5 May 2010	28 July 2011	8	13	14	23	7	11	6	10	6	10	4	7	0	0	16	26	61	100
Salbutamol^2^	R03AC02	21 December 2011	26 March 2008	10	38	7	27	0	0	1	4	2	8	0	0	2	8	4	15	26	100
Roflumilast^1^,^2^	R03DX07	17 January 2012	11 April 2012	8	19	0	0	2	5	8	19	8	19	9	21	2	5	5	12	42	100
Omeprazole	A02BC01	24 October 2011	20 January 2012	48	87	0	0	0	0	2	4	0	0	0	0	0	0	5	9	55	100
Esomeprazole^2^	A02BC05	19 January 2012	20 January 2012	53	34	0	0	0	0	1	1	0	0	0	0	100	65	0	0	154	100
Rabeprazole	A02BC04	27 April 2011	20 May 2011	21	38	10	18	0	0	0	0	9	16	16	29	0	0	0	0	56	100
Diclofenac	M01AB05	30 June 2011	23 February 2011	50	43	0	0	7	6	16	14	12	10	6	5	9	8	16	14	116	100
Celecoxib^2^	M01AH01	17 November 2010	4 February 2011	78	46	12	7	0	0	5	3	3	2	16	10	15	9	39	23	168	100
Ketorolac	M01AB15	7 June 2011	10 March 2005	17	85	0	0	0	0	1	5	0	0	2	10	0	0	0	0	20	100
Zolpidem	N05CF02	1 October 2010	14 April 2010	13	7	41	21	1	1	7	4	1	1	1	1	84	42	52	26	200	100
Olanzapine^1^	N05AH03	11 January 2012	21 June 2011	37	33	15	14	0	0	2	2	1	1	3	3	8	7	45	41	111	100
Alprazolam	N05BA12	22 August 2007	23 August 2011	61	70	12	14	1	1	0	0	6	7	3	3	3	3	1	1	87	100
Aripiprazole^1^,^2^	N05AX12	20 March 2012	22 February 2012	29	22	9	7	0	0	2	2	0	0	1	1	12	9	79	60	132	100
Paliperidone^1^,^2^,^3^	N05AX13	8 December 2011	6 April 2011	46	58	24	30	0	0	4	5	2	3	1	1	2	3	0	0	79	100
Asenapine^1^,^2^	N05AH05	8 November 2011	11 October 2011	23	38	15	25	0	0	1	2	2	3	0	0	2	3	17	28	60	100
Sum				1398	35	476	12	48	1	198	5	144	4	181	5	792	20	766	19	4003	100
Sum								*n*		%		*n*		%		*n*		%			
ADRs listed with frequency difference or only listed in one country								246		6		325		8		1558		39		4003	100
Sum				*n*		%		*n*						%							
Consistency and discrepancy of ADRs				1874		47		2129						53						4003	100

1 Drug approved via EMA central procedure.

2 Drug approved year 2000 or later.

3 ADR information reasonably assumed to be based on the same clinical study in both countries and highest approved dose is identical.

Percentages may not sum to 100, because of rounding.

### Data analysis of selected variables

We compared the approval agency, the approval date, and the reported underlying clinical studies by comparing the ADR distributions. The approval agency comparison was between the EMA and the DMA, where we used SPCs available from DMA and EMA. Drugs approved before year 2000 was compared to drugs approved year 2000 or later. In the analysis of the first marketing date, the year 2000 was chosen as the European Commission guideline on the SPC structure (European Commission 1999) was noticed to applicants in December 1999. The newest guideline was released in 2009 (European Commission 2009) but few drugs only have been approved under this guideline. We also compared PIs where the ADR information was based on the same underlying clinical study to where this could not be identified. In the analysis of the underlying clinical study, we only considered information stated in the ADR section. The same study was determined from either having the same number of participants in the studies or the same study name. We did not distinguish between studies leading to drug approval and postauthorization studies as in most cases it is not possible to separate the two study types in the PIs.

### Statistical analyses

We used a statistical approach to investigate if the ADRs distributions differed by approval agency, approval date, or clinical study used as base for the information. Initially a chi-square test was performed on the two distributions for each of the three previously stated variables. If this showed a *P*-value less than 0.05, each of the eight categories that ADRs could be assigned to were tested against all the other categories using Fisher’s exact test to identify the category contributing to the difference. In addition to investigating the eight categories, we also investigated if the distributions of *consistent ADRs* and *discrepant ADRs* were significantly different. The category that produced the lowest *P*-value when tested against all other categories was designated the contributing category and excluded from further analysis. The procedure was iterated with a new chi-square test, subsequent Fisher’s exact tests, and exclusion of a category until no significant difference between the distributions was found. Throughout the calculations, Bonferroni correction was used to correct the significance level for multiple testing.

## Results

### Drug selection

**Figure 1:**
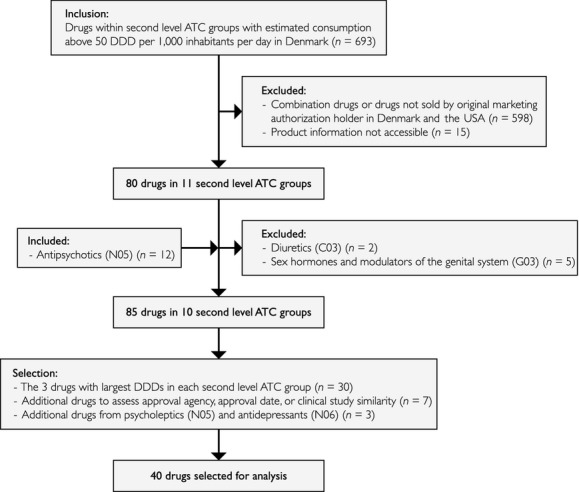
Flowchart of the procedure to select the analyzed sample. Drug selection was based on the therapeutic groups at the second ATC level with DDDs over 50 per 1000 inhabitants per day. The procedure resulted in 40 drugs being selected and analyzed.

Based on the selection criteria (Figure 1), only two *diuretics* (*C03*) were eligible, but these were excluded as they only had a DDD of 0.1 and 0.0 per 1000 inhabitants per day. The group *sex hormones and modulators of the genital system* (*G03*) was also excluded as this group mainly consists of combination drugs and the DDDs of the remaining drugs were low. The group *antipsychotics* (*N05*) and *antidepressants (N06)* were included due to the rapidly increasing consumption of these drugs in Denmark. This resulted in 85 drugs which were reduced to 40 drugs spanning across ten therapeutic groups by selecting the three drugs with largest estimated DDD consumption in each second level ATC group. Additional drugs that could be used to assess if the approval agency, approval date, or clinical study similarity influenced the results were also added.

### Identified ADRs

The PIs for the 40 drugs listed totally 4003 ADRs (Table 2), which were assigned into one of the eight categories of consistency or discrepancy. ADRs listed with *corresponding frequencies* made up 1398 (35%) of all listed. Adding to this category, *corresponding frequencies cannot be dismissed* resulted in 1874 (47%) consistency between Denmark and the USA. The remaining 2129 (53%) showed discrepancy between PIs in the two countries. Among the discrepant ADRs, the largest part was *ADRs only listed in the USA* that made up 1558 (39%), whereas *ADRs only listed in Denmark* constituted 325 (8%). *ADRs listed with frequency difference* represented 246 (6%).

### Consistency and discrepancy

**Figure 2:**
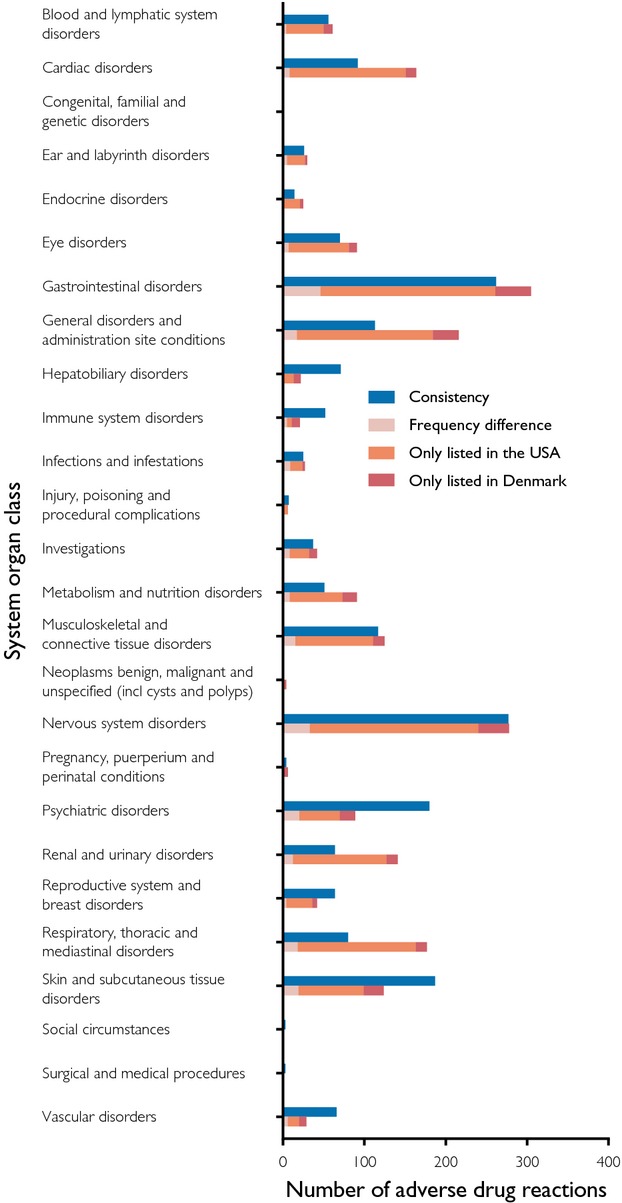
Adverse drug reactions by system organ classes. All consistent and discrepant ADRs for all analyzed drugs divided into SOC classes. The first column of each SOC lists the consistent ADRs. The second column of each SOC lists the discrepant ADRs and the cause of discrepancy.

**Figure 3:**
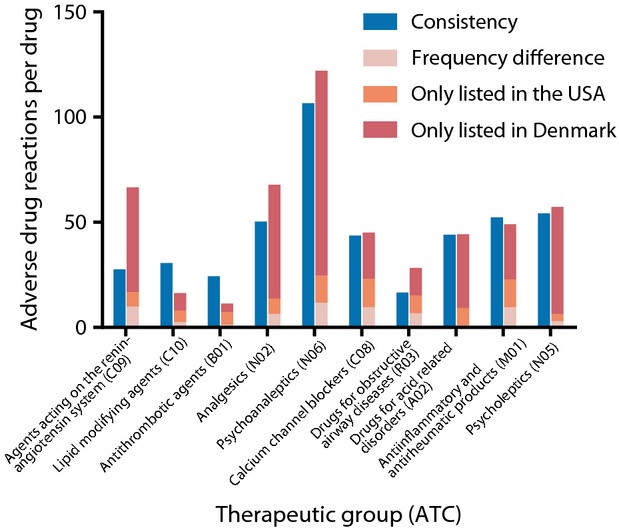
Adverse drug reactions per therapeutic group. Number of ADRs per drug in the ten therapeutic groups analyzed. Therapeutic subgroups are listed in descending order of DDDs per 1000 inhabitants per day in Denmark with second level ATC code in parenthesis.

We identified all ADRs listed in both countries (Table 3). The largest proportion was found in the 673 ADRs with unknown frequency in both countries. The largest proportion of the 2129 discrepant ADRs was the 1558 (73%) *ADRs only listed in the USA*. Of these, 326 were listed as frequent, 440 as infrequent, 532 as rare, and 260 as unknown. Among the 325 (15%) *ADRs only listed in Denmark*, two were listed as very common, 48 as common, 131 as uncommon, 76 as rare, 21 as very rare, and 47 as not known. The ADRs identified as missing in one of the countries span a broad range from less serious (e.g., flushing) to severe reactions (e.g., pulmonary embolism). The remaining 246 (12%) of the 2129 *discrepant ADRs* were *ADRs listed with frequency difference* of which *noncontiguous frequency differences* accounted for 48 (2%). Consistencies and discrepancies were assigned to the affected SOC (Figure 2). The largest numbers of both consistent and discrepant ADRs were found in the SOCs *gastrointestinal disorders* and *nervous system disorders*. We found *antidepressants* (*N06*) to have more listed ADRs than other therapeutic groups (Figure 3).

**Table 3:** Distribution of adverse drug reactions listed in production information issued in Denmark and the USA by frequency

the USA\Denmark	Very common (≥1/10)	Common (≥1/100 to <1/10)	Uncommon (≥1/1000 to <1/100)	Rare (≥1/10,000 to <1/1000)	Very rare (<1/10,000)	Not known
Frequent (≥1/100)	**559**		103	23	3	24
Infrequent (<1/100 to ≥1/1000)	0	31	**294**	53	20	47
Rare (<1/1000)	0	2	11	**99**		32
Unknown	4	29	58	49	6	673*

* ADR listed as identified in post marketing in the USA and stated in Denmark (400), listed with terminology or intervals that do not allow determining if they are consistent neither can they be assigned into any other category (227), or unknown frequency in both countries (46).

Consistent frequencies are marked with bold.

We further determined and compared the distribution of ADRs based on approval agency, approval date, and the reported clinical study (Table 4). Testing for differences between the distributions between EMA and DMA demonstrated a significant difference (*P* < 0.001). The categories contributing to the difference were *ADRs only listed in the USA as rare or unknown* (*P* < 0.001) and *ADRs listed with contiguous frequency difference* (*P* = 0.002). There was no significant difference between *consistent ADRs* and *discrepant ADRs* when comparing the approval agencies (*P* = 0.041). We identified a difference in the distributions between drugs approved before year 2000 and the ones in year 2000 or later (*P* = 0.003). The category contributing to the difference was *ADRs listed with corresponding frequency* (*P* = 0.002). No significant difference was found between *consistent ADRs* and *discrepant ADRs* (*P* = 0.166). Further, we found a significant difference in the distributions between ADR sections based on the same clinical study and those that were not (*P* < 0.001). Here, the categories contributing to the difference were *ADRs only listed in the USA as frequent or infrequent* (*P* < 0.001) and *ADRs only listed in the USA as rare or unknown* (*P* < 0.001). Additionally, the distributions of *consistent ADRs* and *discrepant ADRs* contributed significantly (*P* < 0.001).

**Table 4:** The distributions of adverse drug reactions depending on approval agency, approval date and clinical study

			Approval agency						Approval date						Clinical study					
			EMA			DMA			Before year 2000			Year 2000 or later (*n* = 14 drugs)			Same clinical study (*n* = 11 drugs)			Not same clinical study* (*n* = 29 drugs)		
			(*n* = 10 drugs) *n* (%)			(*n* = 30 drugs) *n* (%)			(*n* = 26 drugs) *n* (%)			*n* (%)			*n* (%)			*n* (%)		
Consistent	ADR listed with corresponding frequency		431 (50)	327 (38)		1443 (46)	1071 (34)		1241 (46)	897 (33)		633 (48)	501 (38)		700 (57)	508 (41)		1174 (42)	890 (32)	
	ADR listed where corresponding frequencies cannot be dismissed			104 (12)			372 (12)			344 (13)			132 (10)			192 (16)			284 (10)	
Discrepant	ADR listed with frequency difference	Contiguous frequency difference	432 (50)	63 (7)	3 (0)	1697 (54)	183 (6)	45 (1)	1454 (54)	159 (6)	39 (1)	675 (52)	87 (7)	9 (1)	532 (43)	83 (7)	9 (1)	1597 (58)	163 (6)	39 (1)
		Noncontiguous frequency difference			60 (7)			138 (4)			120 (4)			78 (6)			74 (6)			124 (4)
												
	ADR only listed in Denmark	Rare, very rare or not known		68 (8)	28 (3)		257 (8)	116 (4)		224 (8)	97 (4)		101 (8)	47 (4)		123 (10)	43 (3)		202 (7)	101 (4)
		Very common, common or uncommon			40 (5)			141 (4)			127 (5)			54 (4)			80 (6)			101 (4)
												
	ADR only listed in the USA	Rare or unknown		301 (35)	85 (10)		1257 (40)	707 (23)		1071 (40)	546 (20)		487 (37)	246 (19)		326 (26)	189 (15)		1232 (44)	603 (22)
		Frequent or infrequent			216 (25)			550 (18)			525 (19)			241 (18)			137 (11)			629 (23)
Sum			863 (100)			3140 (100)			2695 (100)			1308 (100)			1232 (100)			2771 (100)		

* Fluvastatin, valsartan, irbesartan, celecoxib were classified as not based on the same study because the highest strength marketed was not the same.

Percentages may not sum, because of rounding.

## Discussion and Conclusions

The novelty in this work is that we have compared ADR information for a large number of drugs across several therapeutic groups. Our findings unveil large inconsistencies between listed ADRs in PIs from Denmark and the USA. To our knowledge, this is the first time individually listed ADRs and their frequencies have been compared between two countries. The results should be considered conservative as we give the benefit of the doubt and, therefore, do not classify all ADRs not displaying *consistent frequency* as discrepancies, but also permit classification, as *corresponding frequencies cannot be dismissed*. However, when these two categories were merged to form *consistent ADRs*, they still only account for 47%. It is not comforting that we found such a large proportion of discrepancies. The findings undoubtedly bring the question about how similar data or even identical data in the cases when referring to the same study can produce so different information under such similar guidelines. This raises concern about the credibility of PIs as a valuable information source on safe drug use.

The largest proportion of discrepancies was explained by ADRs only listed in one country and this could be considered rather problematic as the information is completely missing in one of the countries. This still occurs although the EMA and FDA PI guidelines are close to identical in respect to presenting ADRs in the PIs. Further, reports of ADRs should be conveyed between markets. Both guidelines state that all ADRs with a causal relationship with the drug in question should be listed. The FDA guideline clarifies this by explicitly stating that reports on ADRs identified both domestically and in foreign countries should be included (Food and Drug Administration 2006; European Commission 2009). It is deeply concerning that we could identify severe ADRs (e.g., pulmonary embolism) in only one country, which should definitely be listed if causality was established. On the other hand, long lists of rare and only possibly causal ADRs could be causing information overload (Silverman 1976; Duke et al. 2011), through what Duke et al. (2011) have labeled “overwarning.” This potentially makes it difficult to distinguish between different drugs’ safety profiles and choosing the most appropriate drug for the patient. Additionally, long lists make the decision harder about what information should be passed on to the patient. The results from this study show similar proportion of consistency as presented by Nieminen et al. (2005). However, it should be pointed out that the results of the two studies are not straightforward comparable as no comparison was made of individual ADRs and only biopharmaceuticals were compared in the study by Nieminen and colleagues. In our study, biopharmaceuticals were not included. In addition, biologicals and small molecule drugs are governed by different legislation (Nieminen et al. 2005). Shimazawa and Ikeda (2013) and Kesselheim et al. (2013) indicated that there were variances in the information provided in different countries. In contrast, our study adds specific comparison of individual ADRs listed for individual drugs between countries. As many clinical trial results are unpublished or reported selectively (Gøtzsche and Jørgensen 2011), we did not compare individual trials with the PIs. It would be desirable to investigate which PI is more close to clinical studies conducted by analyzing raw data (Barbui et al. 2011). This might become reality as EMA has flagged for its commitment to publish clinical trial information (Cohen 2013) and thereby allow public investigation.

We argue that it cannot in any way be beneficial that healthcare providers have access to different medical information and we, therefore, stress the particular value of further standardizing ADR presentation. We propose a combination of the current PI layouts from Europe and the USA, where in similar fashion as in Denmark, a list of aggregated ADRs and their frequencies are presented in a general table. This section could be followed by more detailed information and specific results from clinical trials like in PIs from the USA, possibly also including information on ADRs with weaker causal links. This would provide the possibility to both get an overview of a drug’s safety profile as well as allowing detailed risk–benefit assessments for each patient. Another possibility to adding the information in the PIs is to separate the latter into a separate resource.

### Strengths and limitations of this study

In this study, we based the investigation on Denmark and the USA. The situation in other EU and non-EU countries might differ, and therefore the ADR labeling status for the selected drugs may not be generalizable to other countries and regulatory systems. Moreover, we included only ADRs from the ADR sections unless this referred to other sections. In spite of statements in both European Commission and FDA guidelines (European Commission 2009; Food and Drug Administration 2013) to list all ADRs in the ADR section, it is possible that ADRs have been listed in other sections of the PI only and, therefore, were not included in the analysis. Further, we cannot rule out that the same study was used in both countries even though in our analysis these have been assigned as not the same study. This is because the MAH might use the same study but never provide information about this in the PIs. The discrepancies might also be due to differences in the legal systems, where in the USA, an MAH might provide unnecessarily many ADRs to prevent or lessen the effect of a legal dispute. However, this has not been investigated.

The approval agency did not significantly affect the level of *consistent ADRs*. San Miguel et al. (2005) investigated interaction information in PIs and found no differences among authorization procedures. Drugs approved before 2000 and therefore marketed for a longer period of time have not converged to a common profile. Despite still having a high proportion of discrepancies, PIs of drugs approved after 2000 showed improved consistency. This could potentially be a result of the introduction of the guideline in 2000, but this has not been investigated. This finding undoubtedly brings the question about how identical data under such similar guidelines are not even close to being identical and raises concern about the credibility of ADR information in PIs from both Denmark and the USA.

Although our findings were robust across the included therapeutic categories, we cannot generalize to all other categories of drugs.

In this study, we showed low consistency between PIs for the same drugs marketed in both Denmark and the USA. This occurred regardless of almost identical PI guidelines in both countries. Currently, the ADR information in PIs most likely is not accurate and up-to-date in any of the two studied countries. This undermines the PIs as the valuable information source on safe drug use that they are intended to be. Regulatory agencies are encouraged to make information about all clinically relevant ADRs available to healthcare providers and patients across countries.

## Abbreviations

ADR: adverse drug reaction
ATC: Anatomical Therapeutic Chemical
DDD: defined daily dose
DMA: Danish Medicines Agency
EMA: European Medicines Agency
FDA: Food and Drug Administration
MAH: marketing authorization holder
PI: product information
SOC: system organ class
SPC: Summary of Product Characteristics

## References

[b1] Alloza J, Lasagna L (1983). A comparison of drug product information in four national compendia. Clin Pharmacol Ther.

[b2] Barbui C, Baschirotto C, Cipriani A (2011). EMA must improve the quality of its clinical trial reports. BMJ.

[b3] Bawazir SA, Al-Hassan MI, Al-Khamis KI, Abou-Auda HS, Gubara OA (1991). Comparative study of Saudi-marketed products and US drug labeling. DICP.

[b4] Brown E, Mann RD, Andrews EB (2007). Medicinal Dictionary for Regulatory Activities (MedDRA). Pharmacovigilance.

[b5] Cohen D (2013). EMA consults public on plan to increase transparency of drug trial data. BMJ.

[b6] Duke J, Friedlin J, Ryan P (2011). A Quantitative Analysis of Adverse Events and “Overwarning” in Drug Labeling. Arch Intern Med.

[b7] Dunne M, Herxheimer A, Newman M, Ridley H (1973). Indications and warnings about chloramphenicol. Lancet.

[b8] European Commission (1999).

[b9] European Commission (2009).

[b10] European Medicines Agency (2012). http://www.ema.europa.eu/ema/index.jsp?curl=pages/medicines/landing/epar_search.jsp.

[b11] Food and Drug Administration (2006).

[b12] Food and Drug Administration (2012). http://www.accessdata.fda.gov/scripts/cder/drugsatfda/index.cfm.

[b13] Food and Drug Administration (2013).

[b14] Garbe E, Andersohn F (2007). Contraindication labelling changes in the USA and Germany. Eur J Clin Pharmacol.

[b15] Gøtzsche PC, Jørgensen AW (2011). Opening up data at the European Medicines Agency. BMJ.

[b16] Kesselheim AS, Franklin JM, Avorn J, Duke JD (2013). Speaking the same language? International variations in the safety information accompanying top-selling prescription drugs. BMJ Qual Saf.

[b17] Lægemiddelstyrelsen (2012a). http://www.medicinpriser.dk.

[b18] Lægemiddelstyrelsen (2012b). http://www.produktresume.dk/docushare/dsweb/View/Collection-96.

[b19] Malinowski HJ, Westelinck A, Sato J, Ong T (2008). Same drug, different dosing: differences in dosing for drugs approved in the USA, Europe, and Japan. J Clin Pharmacol.

[b20] Nieminen O, Kurki P, Nordström K (2005). Differences in product information of biopharmaceuticals in the EU and the USA: implications for product development. Eur J Pharm Biopharm.

[b21] Prescrire (2013). 2012 drug packaging review: many dangerous, reportable flaw. Prescrire Int.

[b22] Reggi V, Balocco-Mattavelli R, Bonati M, Breton I, Figueras A, Jambert E (2003). Prescribing information in 26 countries: a comparative study. Eur J Clin Pharmacol.

[b23] San Miguel MT, Martínez JA, Vargas E (2005). Food-drug interactions in the summary of product characteristics of proprietary medicinal products. Eur J Clin Pharmacol.

[b24] Sasich LD, Barasain MA, Al Kudsi MA (2009). Three country comparison of selected safety information in the prescribing information for rosiglitazone (Avandia). Saudi Pharm J.

[b25] Sawalha AF, Sweileh WM, Zyoud SH, Jabi SW (2008). Comparative analysis of patient package inserts of local and imported anti-infective agents in Palestine. Libyan J Med.

[b26] Shimazawa R, Ikeda M (2013). Safety information in drug labeling: a comparison of the USA, the UK, and Japan. Pharmacoepidemiol Drug Saf.

[b27] Silverman M (1976). The drugging of the Americas. How multinational drug companies say one thing about their products to physicians in the USA, and another thing to physicians in Latin America.

[b28] Statens Serum Institut (2012). http://www.medstat.dk.

[b29] Sukkari SR, Al Humaidan AS, Sasich LD (2012). The usefulness and scientific accuracy of private sector Arabic language patient drug information leaflets. Saudi Pharm J.

[b30] WHO Collaborating Centre for Drug Statistics Methodology (2012). Guidelines for ATC classification and DDD assignment, 2013.

